# Brain gene expression analyses in virgin and mated queens of fire ants reveal mating‐independent and socially regulated changes

**DOI:** 10.1002/ece3.3976

**Published:** 2018-04-02

**Authors:** Travis L. Calkins, Mei‐Er Chen, Arinder K. Arora, Chloe Hawkings, Cecilia Tamborindeguy, Patricia V. Pietrantonio

**Affiliations:** ^1^ Department of Entomology Texas A&M University College Station TX USA; ^2^ Department of Entomology National Chung Hsing University Taichung City Taiwan; ^3^ Department of Entomology Cornell University Ithaca NY USA

**Keywords:** abaecin, astakine, G‐protein‐coupled receptor, hexamerin, juvenile hormone, reproductive maturation

## Abstract

Transcriptomes of dissected brains from virgin alate and dealate mated queens from polygyne fire ants (*Solenopsis invicta*) were analyzed and compared. Thirteen genes were upregulated in mated queen brain, and nine were downregulated. While many of the regulated genes were either uncharacterized or noncoding RNAs, those annotated genes included two hexamerin proteins, astakine neuropeptide, serine proteases, and serine protease inhibitors. We found that for select differentially expressed genes in the brain, changes in gene expression were most likely driven by the changes in physiological state (i.e., age, nutritional status, or dominance rank) or in social environment (released from influence of primer pheromone). This was concluded because virgins that dealated after being separated from mated queens showed similar patterns of gene expression in the brain as those of mated queens for *hexamerin 1*,* astakine*, and *XR_850909*. *Abaecin* (*XR_850725*), however, appears upregulated only after mating. Therefore, our findings contribute to distinguish how specific gene networks, especially those influenced by queen primer pheromone, are regulated in queen ants. Additionally, to identify brain signaling pathways, we mined the fire ant genome and compiled a list of G‐protein‐coupled receptors (GPCRs). The expression level of GPCRs and other genes in the “genetic toolkit” in the brains of virgin alates and mated dealate queens is reported.

## 1 INTRODUCTION

The red imported fire ant (*Solenopsis invicta* Buren) relies on reproductive queens to produce eggs, and completely sterile female workers devoid of ovaries (Khila & Abouheif, 2008) to forage, rear brood, defend the colony, and perform other nest‐maintaining tasks. This invasive species has two genetically determined social forms, monogyne, in which colonies have a single mated queen, and polygyne, in which multiple mated queens cooperate (Ascunce et al., 2011). In a mature polygyne colony, dealate mated queens produce eggs while suppressing the reproductive maturation of alate virgin queens through an as yet unidentified primer pheromone (Fletcher & Blum, 1981). Virgin queens require a period of reproductive maturation in the nest before they can engage in a mating flight (Lofgren, Banks, & Glancey, 1975). Concomitant with their reproductive status, these two types of queens differ in their behavior and physiological states. Mated queens regulate colony social organization and growth through dominance by releasing primer pheromone that influences the physiology and behavior of workers and other reproductive adults (Vargo, 1999; Vargo & Laurel, 1994; Vinson, 1997). The mated queen primer pheromone that is distributed among members of the colony suppresses the corpora allata (CA) activity of virgin queens, resulting in low juvenile hormone (JH) titer that prevents virgin queens’ ovarian development, impeding their reproduction (Fletcher & Blum, 1981). The queen primer pheromone also prevents alate virgin queens inside the colony from shedding their wings. However, alate virgin queens shed their wings and their ovaries develop after being experimentally removed from queen pheromonal influences (Fletcher & Blum, 1981, 1983; Vargo, 1999), coinciding with global changes in gene expression as reported with queen whole bodies (Wurm, Wang, & Keller, 2010). Further, in this scenario, when several alate queens are grouped, those that dealate first exhibit dominance, preventing the remaining alates from dealation (Fletcher, Cherix, & Blum, 1983).

The central neuroendocrine system of queens undoubtedly plays a crucial role in regulating the above‐described pheromone‐driven, mating‐associated changes and social organization processes, as described for honey bees (Kocher, Richard, Tarpy, & Grozinger, 2008). Gene expression changes in queen brains/retrocerebral complex (CA and corpora cardiaca) and ovaries are expected not only after mating but also with changes in social context or nutritional status of queen and colony, which certainly affects reproductive output.

In fire ants, we previously focused on characterizing some of the players in the genetic conserved toolkit (Chen, Lewis, Keeley, & Pietrantonio, 2004; Lu & Pietrantonio, 2011b; Lu, Vinson, & Pietrantonio, 2009; Toth & Robinson, 2007; Vinson, Pietrantonio, Lu, & Coates, 2008). Additionally, some of our pioneering work focused on a G‐protein‐coupled receptor (GPCR) signaling system, the sNPF (sNPY) system. We showed that mated queen starvation resulted in the significant reduction in *sNPF receptor* transcripts in queen brains suggesting this system links nutrition and reproduction (Ament, Velarde, Kolodkin, Moyse, & Robinson, 2011; Bajracharya, Lu, & Pietrantonio, 2014; Chen & Pietrantonio, 2006). Importantly, the sNPF receptor protein is present in the brain of both virgin and mated queens but it is only expressed in oocytes in ovaries of mated queens (Lu & Pietrantonio, 2011a). Bai and Palli (2016) recently reported that *sNPF receptor* silencing impairs vitellogenin uptake in red flour beetle oocytes, further linking sNPF signaling system with nutritional status and reproductive output. The sNPF receptor occurs in cell clusters in brains of both mated and alate queens (Lu & Pietrantonio, 2011a), and it is differentially expressed in the brain of different worker subcastes depending on task performed and importantly, depending on the presence or absence of larvae and eggs in the colony (Castillo & Pietrantonio, 2013). Larvae in the 4th instar are the only ones able to digest solid protein (Petralia, Sorensen, & Vinson, 1980). These changes in the protein expression of the sNPF receptor in reproductive and worker castes likely reflect the colony integration of nutritional signals for colony growth (Castillo & Pietrantonio, 2013).

Similarly, transcriptional changes in the brains of queens reflective of the physiological changes occurring in queens’ transition from virgin alates to egg‐laying queens after the mating flight are expected. As such, we aimed to identify differences in brain gene expression between mated and virgin queen fire ants, which is a current gap in our understanding of sexual maturity, queen dispersal, mating, and postmating events leading to their ecological success. Moreover, molecular mechanisms of other physiological adaptations for mating or nest‐living may be revealed by analyzing these transcriptomes. For example, the size of carpenter ant brain decreases after the mating flight, due to reduction in the size of visual neuropiles as queens transition to live permanently underground in darkness (Julian & Gronenberg, 2002).

In this study, we investigated changes in transcriptomes between alate virgin and dealate mated queen brains. From this dataset, we aimed to (1) identify differentially expressed genes (DEGs) among these two conditions; (2) compile the currently annotated GPCR genes in the fire ant genome (Wurm et al., 2011) and identify those GPCRs transcripts expressed in the fire ant brain that may contribute to relevant signaling networks in queens, as we showed for the sNPF receptor. Further, we investigated whether the level of expression of the validated genes identified as differentially expressed by transcriptome analyses changed in newly mated queens and in virgin queens held in queenright or queenless conditions; (3) we tested the hypothesis that expression of *hexamerin‐like* mRNA (hereafter designated *hexamerin 1*; XM_011157206, Table 1) in virgin queens is controlled by JH by applying a JH mimic.

**Table 1:** Differentially expressed genes in brains of dealate mated versus alate virgin queens

Gene ID	Gene name	Fold change^a^	Total FPKM^b^	Gene description *S. invicta* c	GO terms (from Ensembl Metazoa^c^)
XM_011157206	LOC105192919	−9	2,983.8	Hexamerin‐like mRNA	
XM_011158637	LOC105193944	−3.04	171.45	Facilitated trehalose transporter Tret1‐like, transcript variant X2, mRNA^d^	GO:0016020: membrane; GO:0016021: integral component of membrane; GO:0008643: carbohydrate transport; GO:0055085: transmembrane transport; GO:0005215: transporter activity; GO:0022857: transmembrane transporter activity; GO:0022891: substrate‐specific transmembrane transporter activity
XM_011171175	LOC105202586	−3.14	159.87	Uncharacterized LOC105202586, mRNA	GO:0004144: diacylglycerol O‐acyltransferase activity
XR_851183	LOC105203692	−4.42	138.31	Uncharacterized LOC105203692 ncRNA	
XM_011174167	LOC105204893	−2.79	92.55	Uncharacterized LOC105204893 partial mRNA	
XM_011160766	LOC105195388	−4.21	62.52	Venom protease‐like partial mRNA	
XM_011161187	LOC105195675	−6.44	45.67	UDP‐glucuronosyltransferase 2C1‐like, transcript variant X2, mRNA	GO:0008152: metabolic process; GO:0016758: transferase activity, transferring hexosyl groups
XM_011157651	LOC105193270	−10.89	24.36	Chymotrypsin‐1‐like mRNA	GO:0006508: proteolysis; GO:0004252: serine‐type endopeptidase activity
XM_011157183	LOC105192898	−7.07	17.42	Arylphorin subunit alpha‐like mRNA (hexamerin‐like)	
XR_850725	LOC105195721	8.71	1,113.4	Uncharacterized LOC105195721, ncRNA (*nc725*; abaecin)	
XR_850909	LOC105199067	2.91	1,078.8	Uncharacterized LOC105199067, ncRNA (*nc909*)	
XM_011167747	LOC105200273	3.63	617.96	Chymotrypsin inhibitor‐like mRNA	
XM_011157536	LOC105193179	6.48	408.9	Endocuticle structural glycoprotein SgAbd‐1‐like mRNA	GO:0042302: structural constituent of cuticle
XM_011157405	LOC105193076	2.75	256.88	Uncharacterized LOC105193076, mRNA	
XM_011167741	LOC105200265	2.79	233.08	Astakine‐like mRNA	GO:0016021: integral component of membrane
XM_011167750	LOC105200276	3.67	95.61	Chymotrypsin inhibitor‐like mRNA	
XM_011165710	LOC105198862	3.68	55.88	Peptide‐methionine sulfoxide reductase, transcript variant X4, mRNA	GO:0055114: oxidation–reduction process; GO:0008113: peptide‐methionine (S)‐S‐oxide reductase activity
XM_011174636	LOC105205292	3.21	33.77	Uncharacterized LOC105205292, mRNA^e^	GO:0006508: proteolysis; GO:0008237: metallopeptidase activity; GO:0008270: zinc ion binding
XM_011161436	LOC105195830	2.48	30.88	Uncharacterized LOC105195830	GO:0006508: proteolysis; GO:0004222: metallo‐endopeptidase activity; GO:0008237: metallopeptidase activity
XM_011164314	LOC105197777	3.98	30.72	Phenoloxidase 2, mRNA	GO:0008152:metabolic process; GO:0016491: oxidoreductase activity
XM_011173486	LOC105204417	4.5	22.67	Membrane metallo‐endopeptidase‐like 1 mRNA	GO:0006508: proteolysis; GO:0007218: neuropeptide signaling pathway; GO:0004222: metallo‐endopeptidase activity; GO:0008237: metallopeptidase activity
XM_011157963	LOC105193507	4.85	15.78	Uncharacterized LOC105193507	

*q*‐values for all differentially expressed genes <0.05.

a Fold change indicates the relative change in expression in mated queen brains compared to virgin alate queens (negative values indicate lower expression in mated queens).

b Fragments per kilobase of exon per million reads mapped.

c NCBI *Solenopsis invicta* Genome Annotation Release 100 (https://metazoa.ensembl.org/index.html).

d We previously cloned this cDNA and reported it as a putative glucose transporter 8 GenBank: AY911645.1 (Chen, Holmes, & Pietrantonio, 2006).

e Blast analyses revealed that this predicted transcript appears to include the sequences of three contiguous aminopeptidases N.

## 2 MATERIALS AND METHODS

### 2.1 Insects

For transcriptomics, polygyne fire ant colonies were field‐collected from 29 June 2016, until August 2016 at the Texas A&M University Campus in College Station (West Campus), Texas. Colonies were dripped and maintained in the laboratory as described (Chen et al., 2004). Brain dissections of alate virgin and dealate mated queens (all of unknown age) from these colonies were performed. Mated queen brains were pooled depending on the success in obtaining mated queens. To obtain sufficient mated queen brains, mated queens (unknown age) were also obtained from polygyne colonies collected in a pecan orchard at the Texas A&M University Farm (30°31′16.07″N; 96°25′30.40″W), and also at the 5 Eagle Ranch (Burleson County, near Caldwell, TX 30°37′49.93″N; 96°40′19.48″W). Two mated queen pools contained brains from colonies collected at TAMU and the pecan orchard, and one of them also included 10 brains from ants at the 5 Eagle Ranch. The other two pools proceeded from the pecan orchard. For validation of DEGs, and for bioassays (alate separation and JH application), colonies containing both dealate mated and alate virgin queens were collected during the late spring and summer of 2017 in the same pecan orchard as indicated above. One or two days after heavy rains, newly mated queens were collected immediately following a mating flight after landing and dealating, when they were easily seen walking on parking lots and sidewalks at Texas A&M University Campus in College Station, at the same location where alates were collected for transcriptomes.

### 2.2 Brain dissections and RNA extraction for transcriptomic analyses

Queen brains were dissected on ice‐cold PBS buffer and immediately placed in 100 μl of TRIzol Reagent (Thermo Fisher Scientific, Carlsbad, CA) on dry ice. Once brains (approximately 35 per tube constituting one sample) were dissected, the TRIzol reagent was thawed and the brains homogenized by vortexing twice for 30 s. After homogenization, 400 μl of TRIzol was added and mixed by inverting the tube. The solution was incubated at room temperature for 10 min. After incubation, 100 μl of chloroform was added, and the solution was mixed thoroughly by inverting the tube for 15 s and incubated at room temperature for 5 min. After centrifugation (13,000 RCF for 30 min at 4°C), the aqueous phase was transferred to a new tube and RNA was precipitated overnight at −20°C using 10 μg RNase‐free UltraPure^™^ glycogen (Thermo Fisher Scientific) and 500 μl of isopropanol. The supernatant was discarded, and the pellet was washed with 500 μl of 75% ethanol by inverting the tube slowly without disturbing the pellet.

Traces of TRIzol from the solubilized pellet were removed using Direct‐zol^™^ RNA MicroPrep kit (Zymo Research, Irvine, CA) columns, including optional DNase I treatment, following the manufacturer's instructions. Any remaining DNA contamination was removed using TURBO DNA‐free^™^ Kit (Life Technologies, Carlsbad, CA). Following elution, the RNA was quantified using as Infinite^®^ 200 NanoQuant (Tecan Trading AG, Switzerland). RNA was then stored at −80°C.

### 2.3 Libraries preparation and sequencing

Four total RNA samples from dealate mated queen brains and four total RNA samples from alate virgin queen brains were submitted to the AgriLife Genomic and Bioinformatic Center for transcriptome sequencing. A minimum of 35 brains were used for each sample that yielded approximately 1 μg of total RNA; from each of these samples, 5 μl (~400 ng) was kept at −80°C for subsequent qRT‐PCR verification. The libraries were prepared with Illumina TruSeq Stranded Total RNA library preparation kit and sequenced with the HiSeq 2500 System (Illumina) in four lanes of 125SE (single end). The sequence raw reads were cleaned using cutadapt 1.0 to remove barcode tags and adaptors. Individual samples were processed with FastQC, and the QC reports were checked as final confirmation of sequence quality.

### 2.4 Bioinformatic data analyses

The processed reads were uploaded to the Discovery Environment web interface and platform at CyVerse (Goff et al., 2011). The RNA‐seq reads that passed the quality filters (FASTQC tools) were mapped to the *S. invicta* genome (Ensembl Metazoa, GCA_000188075.1.34) using TopHat 2.0.9 (Trapnell, Pachter, & Salzberg, 2009). Cufflinks 2.1.1 was used to estimate the transcript abundance (Trapnell et al., 2010). We performed two types of analyses. First, DEGs were identified using Cuffdiff 2.2.1 with JS (Jensen–Shannon) option (Trapnell et al., 2012). The identification of DEGs was performed with the default false discovery rate (FDR = 0.05). DEGs were researched and annotated using UniProt Knowledgebase (Bateman et al., 2015) and Blast similarity searches and reciprocal searches at NCBI. All sequence data have been submitted to the GenBank databases NCBI GEO under accession number (GSE108063: Fire ant alate virgin and dealate mated queen brain transcriptomes).

Second, we focused our attention to the expression of a small number of genes generally involved linking reproduction, nutrition, growth, and division of labor, known as the “genetic toolkit” (Toth & Robinson, 2007). For ants, these were identified by Okada, Watanabe, Tin, Tsuji, and Mikheyev (2017). To identify putative *S. invicta* GPCR genes, blastP searches in NCBI using identified GPCRs from other insect species as listed by Caers et al. (2012) were performed. We also searched NCBI using “GPCR *Solenopsis invicta*” as keywords. The compilation of GPCRs included only candidates in which the predicted 7tm_GPCRs superfamily Conserved Protein Domain Family was identified by NCBI. Additional GPCRs, in which the transmembrane regions were wrongly predicted, were included in the compilation after TMpred analyses (expasy.org; Hofmann & Stoffel, 1993) verified the presence of seven transmembrane domains. We additionally identified those GPCRs that were most abundant in the brain transcriptome (FPKM > 10) and compiled a list of their relative levels of expression.

### 2.5 Quantitative RT‐PCR

All cDNAs used for quantitative RT‐PCR (qPCR) in this study were synthesized from 140 ng of total RNA using the SuperScript^®^ III First‐Strand Synthesis System (Thermo Fisher Scientific) following the manufacturer's protocol.

Two genes, *elf1‐beta* and *rpl18* (Cheng, Zhang, He, & Liang, 2013), were used as reference (primer sequences in Table S1). Each reaction contained 5 μl PowerUp Sybr^™^ master mix (Applied Biosystems), primers (300 nmol/L each applied as 1 μl of 3 μmol/L solution; Sigma‐Aldrich), and 5 ng cDNA. The volume was adjusted with nuclease‐free water to 10 μl. Reactions were prepared and run with two technical replicates for each gene of interest (GOI) and cDNA sample, and four biological replicates were performed. The following thermocycling program in a QuantStudio Flex 6 qPCR thermocycler (ThermoFisher) was used 3 min at 95°C, followed by 40 cycles of 95°C for 15 s and 60°C for 30 s. Primer specificity was monitored with “melt curve” analysis in the QuantStudio 6 program. Data were analyzed via the ∆∆Ct method utilizing both reference genes (Vandesompele et al., 2002). Statistical differences were determined by comparing ∆Cts of GOIs between samples with unpaired *t* test or ANOVA followed by posthoc Tukey's test, using GraphPad Prism 6 software (GraphPad Software Inc., La Jolla, CA).

### 2.6 Transcriptome verification and validation by qRT‐PCR of selected genes

For verification and validation of mRNAs differentially expressed between dealate mated and alate virgin queens, four GOI—two predicted coding transcripts, *hexamerin 1* and *astakine*, and two predicted noncoding mRNAs, *XR_850725* and *XR_850909* (hereafter designated *nc725* and *nc909*, respectively)—were selected for quantitative qPCR analyses (Table S1). These genes were chosen for analyses due to their high FPKMs values and relevant functions; for example, specifically *hexamerin 1* (*hexamerin‐like*) for relevance to the ant toolkit (Okada et al., 2017). Verification encompassed qPCR analyses that utilized cDNAs synthesized from the remnant (~400 ng) total RNA submitted for sequencing, whereas, validation included qPCR analyses performed with cDNA synthesized from brains from a separate set of queens. For validation analyses, we independently collected colonies during the summer of 2017 from which dealate mated queens and virgin alate queens of unknown age were obtained. Brains were dissected as described in pools of 10 for each of four replicates for both, dealate mated and virgin alate queens, respectively. Similarly, four pools of 10 brains each were obtained from newly mated dealate queens from mating flights that were collected upon landing after mated as described (Lu et al., 2009). Total RNA extraction, cDNA synthesis, and qPCR analyses were as described above.

### 2.7 Virgin alate queen separation assay

To test whether changes in brain gene expression of the four GOIs observed in newly mated/mated queens were a consequence of “mating” or alternatively, occurred as a result of other physiological changes associated with their social environment [separation from the influence of primer pheromone of mated queens; increase in JH (Vargo & Laurel, 1994)], an alate virgin queen separation assay was performed. For this, 12 alate virgin queens were placed either into a plastic container with only workers (0.5 g, ~385 individual workers) (queenless condition) or into a container holding two polygyne dealate mated queens and workers (0.5 g) (queenright condition). Nine replicates (experimental colonies) for each condition were set up in this manner and given daily one fresh cricket and access to 10% honey water ad libitum; a water tube plugged with cotton was placed in each container to provide a moist refuge for queens. Virgin queen dealation was monitored twice daily at 8 a.m. and 8 p.m. Five days after their introduction into these experimental colonies, the brains of virgin queens were dissected as described in “Dissection and RNA extraction.” For each colony condition, brains were pooled for RNA extraction as follows: those of virgins that did not dealate in the queenless colonies (four pools of 10 brains each), those from the same that dealated (four pools of at least seven brains each; this is due to the low number that dealated in queenless conditions), and brains from virgin alates from queenright colonies (four pools of 10 brains each); brains from dealate virgins in queenright colonies could not be obtained due to lack of dealation when inhibited by mated queen pheromone. cDNA was synthesized, and gene expression levels were measured by qPCR; data were subjected to ANOVA as described in “Quantitative RT‐PCR.” Ovaries were also dissected from these virgins for visual comparisons as previously described (Lu et al., 2009).

### 2.8 Juvenile hormone mimic study

Application of a JH mimic S‐hydroprene to virgin alate queens was performed following the protocol by Vargo and Laurel (1994), where queens were treated topically once on the abdomen with 1 μl of S‐hydroprene (Sigma‐Aldrich, St. Louis, MO) (25 ng/μl dissolved in 80% acetone and 20% ethanol), or with 1 μl of a solvent (80% acetone and 20% ethanol; solvent control). Treated queens were introduced back into their colony of origin in a meshed container (separated from other queens or workers but with physical contact being possible) for 12 hr. At this time, it was verified all had dealated, therefore, they were immediately flashed‐frozen in liquid nitrogen and stored at −80°C. Total RNA purification from whole bodies was performed as described above, as well as qPCR analyses for *hexamerin 1* expression.

## 3 RESULTS

### 3.1 Transcriptome description

All queen brain RNA samples obtained with the described protocol were of high quality for sequencing. The number of sequenced reads per sample and the percentage that mapped to the genome are shown in Table S2. Twenty‐two genes were differentially expressed (*q‐*value ≤0.05; with a fold change ≥2) between dealate mated and alate virgin queen brains (Table 1). In total, nine genes were annotated as uncharacterized in the genome (Ensembl Metazoa) but three had associated GO terms indicating molecular function (Table 1).

Of the DEGs, nine genes had significantly lower expression in dealate mated queens. Among them, potentially involved genes in energy‐related processes were two hexamerin genes (*hexamerin 1* [XM_011157206], and *arylphorin subunit alpha‐like*), one uncharacterized protein with a diacylglycerol O‐acyltransferase activity GO term, and the facilitated *trehalose transporter 1‐like*,* Tret1* gene. Transcripts for two putative proteases and one transcript with potential role in metabolic detoxification (UDP‐glucuronosyltransferase *2C1‐like* enzyme), were also downregulated in mated queens. Among the upregulated genes in mated queens were two predicted serine protease inhibitors, an endopeptidase with a GO term for neuropeptide signaling pathway, and two proteins likely involved in hematopoiesis and/or immunity, *astakine* and *phenoloxidase 2* (Table 1).

### 3.2 Transcriptome verification

**Figure 1:**
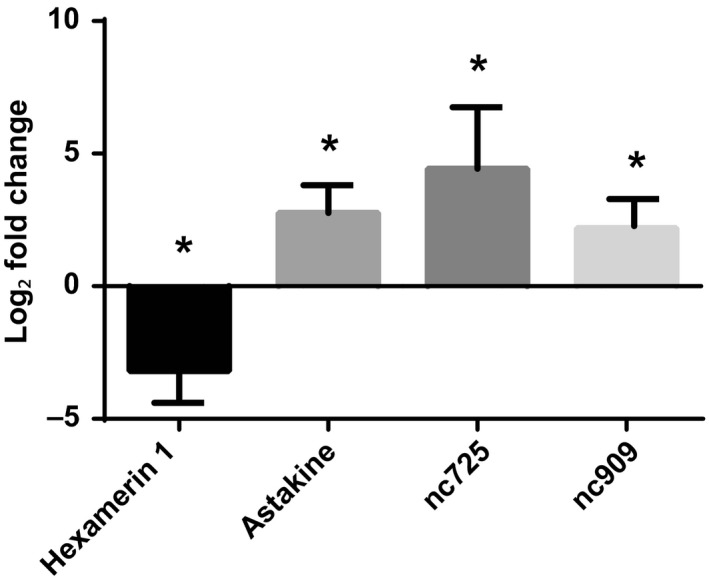
Verification of gene expression of four gene of interests found differentially expressed between mated versus alate queen brains in the transcriptome analysis. cDNA was synthesized from the same total RNA from the four replicates that were sequenced. Gene expression determined by qPCR was normalized to both *elf1‐beta* and *rpl18*. Bars represent mean log_2_ fold change ±SEM. “*” Indicates significant differences between virgin alate and mated queen brains determined by *t* test, *p *<* *.05, *n* = 4. Only *hexamerin 1* gene was downregulated in dealate mated queen brain. *p* Values: *hexamerin 1*, .008; *astakine*, .009; *nc725*, .026; *nc909*, .017

From the genes differentially expressed in the transcriptome, we chose four for quantitative qPCR analyses: *hexamerin 1*,* astakine‐like protein* (*astakine*), and two predicted noncoding RNAs, XR_850725 and XR_850909 (*nc725* and *nc909*, respectively). Analyses of selected GOIs showed significant changes (*p *<* *.05) in expression between alate virgin and dealate mated queen brains for the four genes. *Hexamerin 1* had relatively lower level of expression while the three other genes had higher levels of expression in dealate mated queen brains (Figure 1).

### 3.3 Transcriptome validation

**Figure 2:**
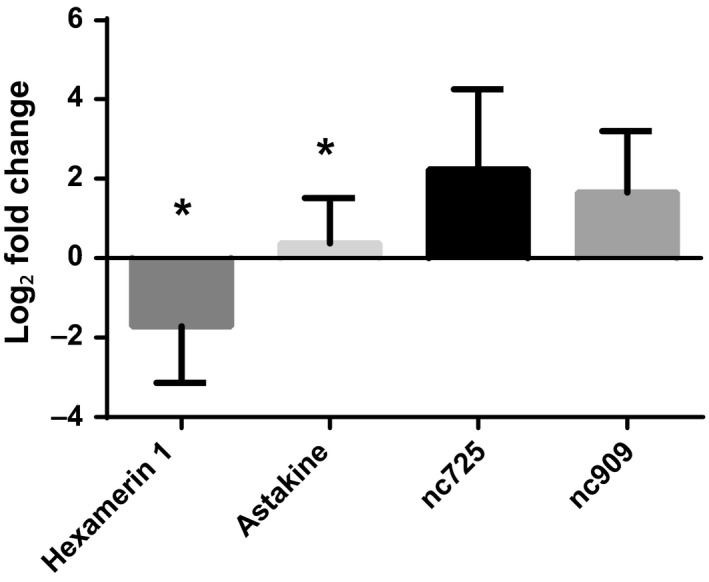
Validation of gene expression with independent samples of four gene of interests found differentially expressed in the transcriptome analysis between mated versus alate queen brains. cDNA was synthesized from independently collected queens. Gene expression determined by qPCR was normalized to both *elf1‐beta* and *rpl18*. Bars represent mean log_2_ fold change ±SEM. “*” Indicates significant differences between virgin alate and mated queen brains determined by *t* test, *p *<* *.05, *n* = 4. *Hexamerin 1* expression was significantly reduced in mated queen brain. The relative trends in gene expression reflect transcriptome results. *p* Values: *hexamerin 1*, .019; *astakine*, .039; *nc725*, .057; *nc909*, .484

To validate the transcriptome results, we utilized qPCR analyses of the same four GOIs as for transcription verification described above, but using as template, the cDNA generated from brains dissected from four subsequently collected independent biological replicates. *Hexamerin 1* was significantly reduced in mated queens (*p *=* *.02), while *astakine* was upregulated (*p *=* *.04) (Figure 2). While the observed trends in gene expression were similar to those of the samples used for obtaining the transcriptome and its verification (Figure 1), the levels of expression of *nc725* (*p *=* *.057) and *nc909* (*p *=* *.4) were not significantly different between dealate mated and virgin alate queen brains (Figure 2).

### 3.4 Analyses of GOIs expression in newly mated queens versus virgin alate queens

**Figure 3:**
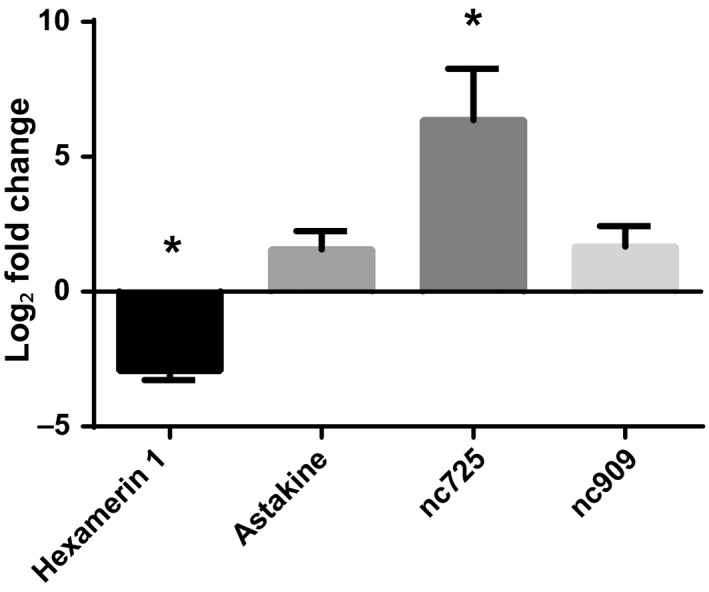
Relative gene expression of the four gene of interests in brains of dealate newly mated (24‐hr postmating flight) queens versus alate virgin queens. Gene expression determined by qPCR was normalized to both *elf1‐beta* and *rpl18*. Bars represent mean log_2_ fold change ±SEM. “*” Indicates significant differences in gene expression between dealate newly mated versus alate virgin queens as determined by *t* tests, *n* = 4. *p* Values: *hexamerin 1*, <.001; *astakine*, .059; *nc725*, .007; *nc909*, .062

qPCR analyses of GOIs expression from newly mated (24‐hr postmating flight) versus alate virgin queens also yielded trends similar to those observed in transcriptome verification (Figure 1) and validation (Figure 2). Additionally, expression of both, *hexamerin 1* and *nc725* (*p *<* *.05), was significantly different, while *astakine* and *nc909* were not (*p *=* *.059 and .062, respectively) (Figure 3).

### 3.5 Virgin alate queen separation assay

To test whether changes in queen brain gene expression were associated with mating or with virgin queen release from the influence of mated queen primer pheromone (disinhibition), we tested the effects of removing virgin alate queens from the presence of mated counterparts. An average of 29.6% of virgin alate queens dealated in queenless conditions by 48 hr of assay initiation, and no further dealation was observed until the assay end point (5 days; Table 2). In a similar setup, conducted in parallel with virgin queens in queenright conditions, no virgin alate queens were dealated (data not shown).

**Table 2:** Number of virgin queens that remained alate or dealated after 48 hr of maintenance in queenless condition. Independent replicates (1–9), each composed of 12 virgin alates in queenless conditions, were set up. Contiguous cells separated by dashed lines correspond to queens which brains were pooled for qRT‐PCR analyses

Replicates	1	2	3	4	5	6	7	8	9
Alate virgin	6	11	4	10	10	9	10	9	7
Dealate virgin (% dealated)	6 50%	1 8.3%	8 66.6%	2 16.7%	2 16.7%	3 25%	2 16.7%	3 25%	5 41.7%

Relative expression of the four GOIs was evaluated by qRT‐PCR in the resulting three groups of virgins. Because of the low number of queens that dealated upon separation from the mated queen, their brains were pooled as follows: qPCR group 1 = alate separation replicates 1 and 2, qPCR group 2 = replicate 3, qPCR group 3 = replicates 4, 5, and 6, and qPCR group 4 = replicates 7, 8, and 9. Similar corresponding pools were assembled for the alate virgins.

**Figure 4:**
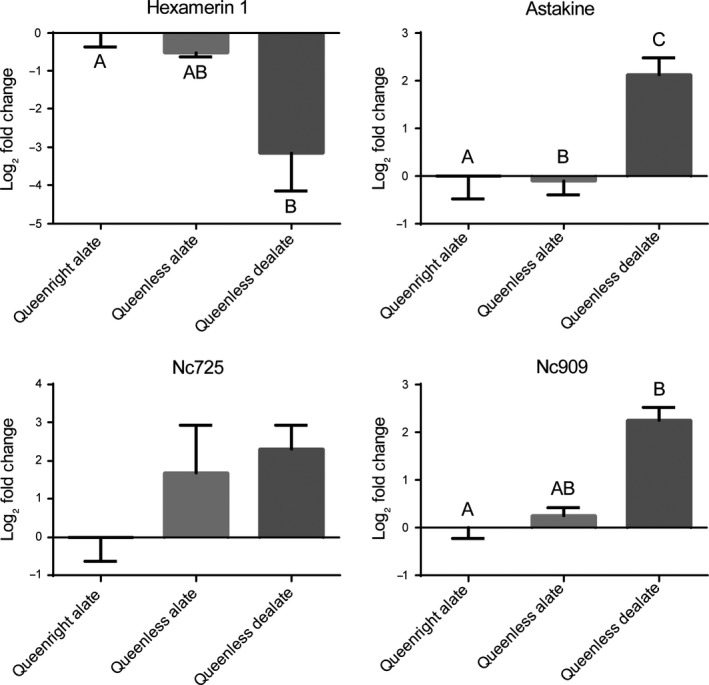
Relative gene expression of four gene of interests in brains of queenright alate, queenless alate, and queenless dealate virgin queens. Gene expression determined by qPCR was normalized to both *elf1‐beta* and *rpl18*. Bars represent mean log_2_ fold change relative to queenright alates ±SEM,* n* = 4. Different letters indicate significant differences in gene expression as determined by one‐way ANOVA and Tukey's posthoc analyses (*p *<* *.05)

**Figure 5:**
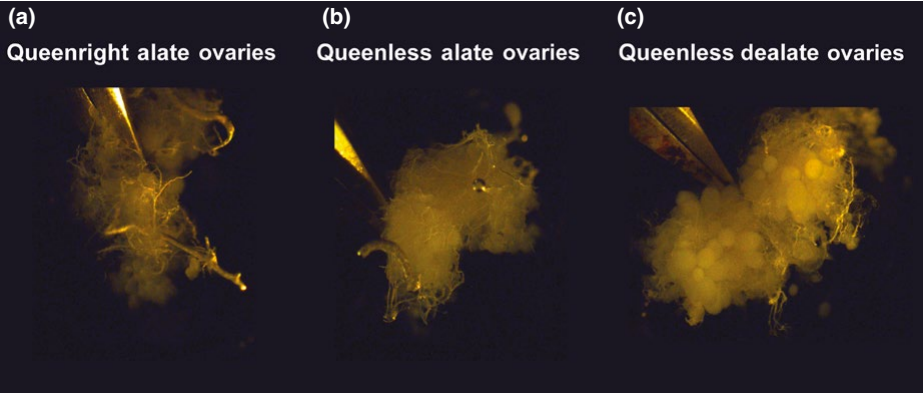
Ovaries of virgin queens 5 days after the initiation of the alate separation assay. (a) Ovaries of virgin alates kept in queenright colonies are small and nonvitellogenic. (b) and (c) Virgins were kept for 5 days in queenless colonies. In (b), ovaries of virgins that did not dealate remained small, similar to those shown in (a) for queenright condition. In (c) virgins dealated within 48 hr of separation from the mated queen and developed vitellogenic ovaries (on the left of each ovary, a pair of forceps holding them is shown)

When comparing queenright alates, queenless alates, and queenless dealates, ANOVA detected significant differences in the relative expression of *hexamerin 1* (*p *=* *.029), *astakine* (*p *<* *.001), and *nc909* (*p *=* *.036). For *hexamerin 1* and *nc909*, significant differences were between queenless dealate and queenright alates (Figure 4). For *astakine*, differences were significant among the three groups. For *nc725*, despite an upward trend in its expression in virgins under queenless condition, no significant differences were detected (*p = *.116; Figure 4). Comparison of ovary morphology of these three groups of virgin females (Figure 5) reflected the suppressive effect of primer pheromone (Fletcher et al., 1983) as expected and quantified previously (Vargo & Laurel, 1994); ovaries were either previtellogenic in inhibited queens showing extremely reduced ovaries (Figure 5a) or vitellogenic from a disinhibited mature virgin queen exhibiting developed oocytes (Figure 5c). In Figure 5b, ovaries with an intermediate phenotype are shown from a virgin queen that did not dealate under queenless condition, with small oocytes, a few vitellogenic.

### 3.6 Juvenile hormone mimic study

**Figure 6:**
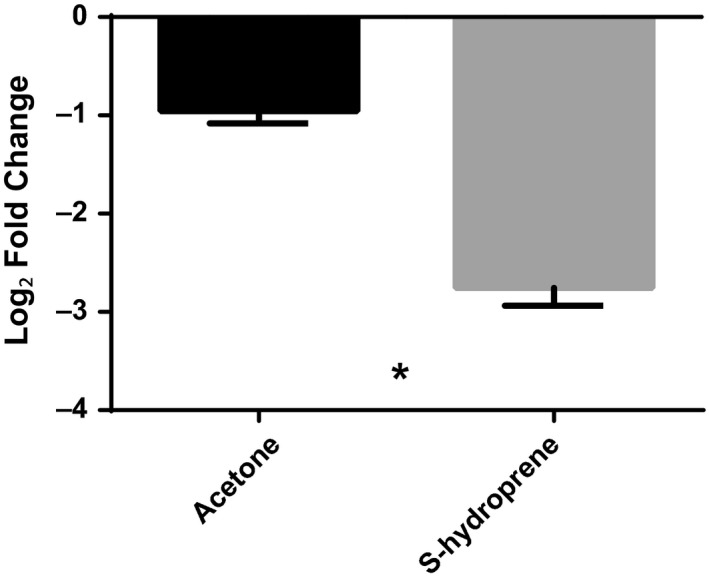
Relative gene expression of *hexamerin 1* in whole bodies of virgin queens treated with the juvenile hormone mimic S‐hydroprene under queenright conditions. Virgin queens dealated under queenright condition when treated with S‐hydroprene; none of the controls (untreated or acetone‐treated) were dealated. Gene expression in whole bodies determined by qPCR was normalized to both *elf1‐beta* and *rpl18*. Bars represent mean log_2_ fold change ±SEM with respect to the nontreated control, *n* = 3 with ten virgins each. “*” Indicates significant difference in gene expression between acetone‐treated control and S‐hydroprene‐treated queens as determined by *t* tests. *Hexamerin 1* was significantly downregulated (*p *=* *.001)

We investigated factors affecting the downregulation of *hexamerin 1* in the dealate virgin queens under queenless conditions (Figure 4) and in dealate mated queens (Figure 1). Virgin alates in queenright conditions were subjected to treatment with a stable JH mimic, S‐hydroprene. All S‐hydroprene‐treated virgins were dealated within 12 hr of application in the presence of the mated queen. Subsequent qPCR analyses performed 12 hr following treatment showed that expression of *hexamerin 1* transcript significantly decreased by about 80% in these virgin dealates in comparison with the untreated and mock‐treated (acetone) virgins (*p *=* *.001) (Figure 6).

### 3.7 Expression of GPCRs and other social insects conserved signaling molecules in brain

None of the GPCRs identified were differentially expressed between mated and virgin queens, and most had very low levels of expression. It is however pertinent to identify those most highly expressed in queen brains (Table 3). These GPCRs included predicted rhodopsin‐like proteins (visual receptors), and biogenic amine GPCRs, such as those for dopamine, serotonin, tyramine, and octopamine. The muscarinic acetylcholine receptor was represented in the list by two isoforms. Among the neuropeptide receptors were those with predicted functions in regulation of diuresis or feeding (calcitonin‐like, diuretic hormone, and tachykinin), the pigment‐dispersing factor receptor (output regulator of circadian clock), allatostatin receptor (involved in JH regulation), and moody GPCR (involved in blood–brain barrier formation). In addition, we compiled a list of GPCRs annotated in the fire ant genome, as they are important signaling molecules currently understudied in ants and such compilation has not been published for fire ants (Table S3).

**Table 3:** G‐protein‐coupled receptors (GPCRs) expressed in queen brains above 10 FPKM. GPCRs are annotated as currently identified in the *Solenopsis invicta* genome and organized from highest to lowest FPKM. A complete list of expressed GPCRs in brains is shown in Table S3

GeneInfo identifier	Protein accession	Transcript accession	Locus	Current annotation	Alate FPKM	Mated FPKM
gi|751222462|	XP_011164467.1	XM_011166165	LOC105199190	Rhodopsin	122.76	322.87
gi|751202946|	XP_011174320.1	XM_011176018	LOC105206526	Dopamine receptor 1	77.99	133.69
gi|751222464|	XP_011164468.1	XM_011166166	LOC105199191	Rhodopsin‐like	91.27	77.23
gi|751236330|	XP_011171996.1	XM_011173694	LOC105204568	PDF receptor	25.29	51.09
gi|751222675|	XP_011164585.1	XM_011166283	LOC105199276	Probable G‐protein‐coupled receptor AH9.1	28.82	35.67
gi|751209716|	XP_011157518.1	XM_011159216	LOC105194339	Probable G‐protein‐coupled receptor 52	25.64	34.81
gi|751223066|	XP_011164802.1	XM_011166501	LOC105199400	Muscarinic acetylcholine receptor DM1 isoform X1	23.83	28.15
gi|751231202|	XP_011169146.1	XM_011170844	LOC105202369	Metabotropic glutamate receptor	25.85	24.04
gi|751205599|	XP_011176389.1	XM_011178087	LOC105208274	Tachykinin‐like peptide receptor 99D, partial	17.37	32.48
gi|751223401|	XP_011164985.1	XM_011166683	LOC105199537	Opsin, ultraviolet‐sensitive‐like	37.04	12.26
gi|751238153|	XP_011173009.1	XM_011174707	LOC105205353	Dopamine receptor 2‐like	22.89	21.39
gi|751212977|	XP_011159291.1	XM_011160989	LOC105195542	G‐protein‐coupled receptor moody isoform X2	17.37	20.21
gi|751212975|	XP_011159290.1	XM_011160988	LOC105195542	G‐protein‐coupled receptor moody isoform X1	20.10	14.75
gi|751220178|	XP_011163208.1	XM_011164906	LOC105198249	Putative Golgi pH regulator C isoform X2	16.06	18.00
gi|751232987|	XP_011170144.1	XM_011171842	LOC105203085	Tachykinin‐like peptide receptor 99D	12.70	18.85
gi|751223068|	XP_011164803.1	XM_011166500	LOC105199400	Muscarinic acetylcholine receptor DM1 isoform X2	14.03	11.49
gi|751233286|	XP_011170315.1	XM_011172013	LOC105203239	Allatostatin‐A receptor‐like isoform X1	13.64	11.55
gi|751219449|	XP_011162809.1	XM_011164507	LOC105197902	Calcitonin receptor‐like	12.55	12.61
gi|751210001|	XP_011157676.1	XM_011159374	LOC105194460	Diuretic hormone receptor‐like	13.13	11.57
gi|751222422|	XP_011164446.1	XM_011166144	LOC105199174	5‐hydroxy‐tryptamine receptor 2A‐like isoform X1	14.54	10.06
gi|751207421|	XP_011156273.1	XM_011157971	LOC105193512	Tyramine receptor 1	9.84	12.28
gi|751223477|	XP_011165026.1	XM_011166724	LOC105199572	5‐hydroxy‐tryptamine receptor 2B‐like	6.77	14.55
gi|751231536|	XP_011169333.1	XM_011171031	LOC105202476	Octopamine receptor beta‐1R‐like	12.93	6.29
gi|751211218|	XP_011158340.1	XM_011160038	LOC105194900	5‐hydroxy‐tryptamine receptor 2A‐like	7.82	10.80

PDF, pigment‐dispersing factor.

Another focus of the transcriptome analysis was the identification in queen brains of expressed conserved genes among social insects that link nutrition and reproduction (Table 4). However, there were no significant differences in the abundance of these transcripts except for the two hexamerins, *hexamerin 1* and *arylphorin subunit alpha‐like*. Of note is that while *vitellogenins 2* and *3* were highly abundant in the mated queen as expected (Corona et al., 2013; Wurm et al., 2011), showing an upward trend in expression with respect to virgin alates, the *vitellogenin receptor* transcript apparently decreased in mated queen brain and was the only one of these genes that had a *q*‐value of 0.08, the closest near significance.

**Table 4:** Social insects conserved genes expressed in queen brains implicated in signaling, and linking nutrition, growth, and/or division of labor

Gene	Locus	Transcript accession	Protein accession	Alate FPKM	Mated FPKM	*q*‐Value
Vitellogenin‐2	LOC105205782	XM_011175285.1	NP_001291513.1	5,154.83	8,001.340	0.999
Vitellogenin‐3	LOC105205783	XM_011175286.1	NP_001291514.1	141.528	6,092.370	0.999
Vitellogenin‐1/4 (this includes both Vg1 and Vg4)	LOC105205865	XM_011175398		237.681	272.394	0.999
Vitellogenin receptor	LOC105200757	XM_011168460.1	XP_011166762.1	18.282	0.41385	0.080
Insulin receptor‐like (InR‐1)	LOC105207962	XM_011177659.1	XP_011175961.1	0.92114	1.01496	0.999
Insulin‐like peptide receptor, transcript variant X2	LOC105195102	XM_011160339.1	XP_011158641.1	3.2725	3.11185	0.999
Insulin‐like peptide receptor, transcript variant X2	LOC105195102	XM_011160338.1	NP_001291521.1	0.36031	0.22321	1.000
sNPF receptor (annotation: prolactin‐releasing hormone receptor)	LOC105195500	XM_011160926	XP_011159228.1	1.74498	2.15420	0.999
sNPF receptor (annotation: prolactin‐releasing hormone receptor)	LOC105195500	XM_011160928	XP_011159230.1	1.67119	0.00067	0.999
sNPF receptor (annotation: prolactin‐releasing hormone receptor)	LOC105195500	XM_011160929	XP_011159231.1	0.676592	1.60495	0.999
sNPF receptor (annotation: prolactin‐releasing hormone receptor)	LOC105195500	XM_011160927	XP_011159229.1	0.000374	1.41590	0.999
sNPF peptide (sNPY)	LOC105194759	XM_011159844.1	XP_011158146.1	1.79674	0.00000	0.999
sNPF peptide (sNPY)	LOC105194759	XM_011159845.1	XP_011158147.1	0.886964	0.00073	1.000
sNPF peptide (sNPY)	LOC105194759	XM_011159846.1		12.3637	13.43440	0.999
sNPF peptide (sNPY)	LOC105194759	XM_011159847.1	XP_011158149.1	22.429	31.67950	0.999
Hexamerin 1	LOC105192919	XM_011157206	XP_011155508.1	3,163.69	328.919	0.032
Hexamerin 2	LOC105204474	XM_011173560	XP_011171862.1	14.0452	3.80346	0.519
Arylphorin‐alpha	LOC105192898	XM_011157183	XP_011155485.1	16.0248	5.75889	0.032
Arylphorin‐beta	LOC105192897	XM_011157182	XP_011155484.1	1.04915	0.820838	0.998

## 4 DISCUSSION

Changes in brain gene expression have been investigated previously by us and others studying social insects, focusing on genes of the “conserved genetic toolkit,” including those involved in nutritional signaling and division of labor among castes or subcastes (Castillo & Pietrantonio, 2013; Daugherty, Toth, & Robinson, 2011; Lu & Pietrantonio, 2011a; Toth et al., 2010). Here we utilized brain transcriptomics to identify genes differentially expressed between mated and virgin queens. Physiological changes in queen brains are expected after a mating flight and mating (Kocher et al., 2008), and here, we investigated this question in an ant species in which queens are the only individuals with reproductive potential in the colony. Therefore, changes in brain gene expression after mating may reflect the following: physiological processes associated with mating or alternatively, reproductive maturation (age, nutrition level), changes in level of aggression or dominance (i.e., virgins separated from primer pheromone influence), or other ecological factors. Although much effort and care were applied to precisely dissect only the queen brains free of other tissues, minute remnants of fat body, trachea, glands, or muscle cannot be completely eliminated. This study focuses on differential gene expression arising from diverse social or ecological context in polygyne fire ants.

The brain transcriptomic analyses identified 22 DEGs between dealate mated and alate virgin queens. Four DEGs *hexamerin‐like*,* astakine*,* nc909*, and *nc725* were selected for qRT‐PCR analyses for both, transcriptome verification and validation. Results from these analyses support the gene expression results obtained with the transcriptome. That is, the same trends in gene expression were shown from additional independently field‐collected biological replicates. This implies that these transcriptome results are ecologically relevant in nature. Here, we delve into the functional significance of the several of the genes identified in this study. The *nc909* is predicted as a long noncoding RNA, and it is a candidate regulatory gene; its role should be further investigated especially with changes in social context and nutrition (Figure 4).

### 4.1 Effect of social context on differential gene expression: Alate separation assay

In virgin queens, the release from the influence of queen primer pheromone (“*disinhibition*”; Fletcher & Blum, 1983) results in dealation, ovary development, and oocyte growth, at least in part mediated by increase in JH (Barker, 1978, 1979). These sexually mature dealate virgin queens begin laying either unembryonated (trophic) eggs or eggs that will develop into haploid males, and these virgin queens are able to suppress the dealation of neighboring virgin queens (Fletcher et al., 1983; Vargo & Laurel, 1994). The results from the alate separation assay are striking in that three of the four genes that were differentially expressed between mated and virgin queens (Figure 1) were also differentially regulated when comparing queenright alates (Figure 4) with dealate virgins (disinhibited). Therefore, it is likely that regulation of these three GOIs relates to the condition of “dealate” queen and perhaps these genes are normally controlled by the influence of mated queen primer pheromone. It is known that separation from queen mated pheromone increases JH in virgin queens (Brent & Vargo, 2003).

Under “queenless treatment,” some virgin queens dealated while others failed to dealate by the assay end point (5 days; Table 2). Those that did not dealate also exhibited suppression of ovary development (Figure 5), presumably by the effect of the virgins in the same colony that dealated earlier, thus, acquiring inhibiting capability. The inhibitory capability of queens has been correlated with high nutritional status. Overwintering queens or those that develop in early spring are less inhibitory due to their lower nutritional reserves (Willer & Fletcher, 1986). In another ant species, the level of aggression has been found responsible for the hierarchical order in achieving high nutritional status (Okada et al., 2017). The inhibitory effect on other virgin queens is predicted to be achieved by females of high nutritional status (Toth, 2017) which have high reproductive potential, as indeed has been observed in fire ants (Willer & Fletcher, 1986). Further, high reproductive status (dominance) is correlated with higher dopamine in the brain, which is a candidate neural target for fire ant queen pheromone (Boulay, Hooper‐Bui, & Woodring, 2001). Dopamine in ants is associated with aggression (Szczuka et al., 2013). Then, it follows that a similar mechanism found by Okada et al. (2017) is operating in fire ants.

### 4.2 Hexamerins

Hexamerins are storage proteins that in holometabolous insects are synthesized in the fat body and subsequently utilized as an amino acid source during metamorphosis (Levenbook & Bauer, 1984). In ants, hexamerins are believed to play an integral role as protein reserves in colony founding (Martinez & Wheeler, 1994; Wheeler & Buck, 1995; Wheeler & Martinez, 1995). As such, the higher expression of *hexamerin 1* in the fire ant alate virgins compared to mated queens is perhaps not surprising (Haisheng, Bradleigh Vinson, & Coates, 2004). In fact, *hexamerin 1* is also the most highly expressed hexamerin in the thorax and abdomen of alate queens (Figure S1). The presence of *hexamerin 1* in the abdomen also suggests that hexamerin 1 is likely involved in amino acid storage to be used in egg production, roles associated with colony founding. What is surprising is that changes in hexamerin gene expression occur in the brain. Additionally, *hexamerin 1* is the only hexamerin detected with relatively high FPKM in the brain (nearly 100 times that of any other hexamerin). This suggests a role for hexamerin 1 beyond that of a storage protein. In fact, multiple hexamerin proteins in the honey bee have been localized to nuclei of ovaries and fat body cells, suggesting a transcriptional regulatory role for these proteins (Martins, Anhezini, Dallacqua, Simoes, & Bitondi, 2011; Martins & Bitondi, 2012, 2016). Hexamerins may also influence caste development in developing bees (Cameron, Duncan, & Dearden, 2013), and soldier differentiation in termites (Zhou, Oi, & Scharf, 2006). Additionally, in a blowfly and grasshopper, hexamerins bind ecdysteroids and JH, respectively (Braun & Wyatt, 1996; Enderle, Käuser, Renn, Scheller, & Koolman, 1983). As such, it is possible that hexamerin 1 plays a similar role in the fire ant brain, either binding hormones or directly participating in gene regulation, contributing to the typical virgin alate phenotype (i.e., contributing to decrease in available JH in their hemolymph). The expression of hexamerins in ant brain has been recently reported associated with nutrient sensing and division of labor (Okada et al., 2017). However, this does not preclude an amino acid storage role of hexamerin 1.

Even as a storage protein, hexamerin 1 may be playing a role in nutritional signaling, as is suggested in termites (Zhou et al., 2006), allowing alates to determine when they are nutritionally ready for a mating flight. While not as highly expressed as *hexamerin 1*, a second hexamerin, *arylphorin subunit alpha‐like*, was also downregulated in the brain transcriptome of mated queens (Table 1). Of the four fire ant hexamerins, arylphorin subunit alpha‐like contains a sequence that is the most similar in the region of the locust hexamerin, which if deleted eliminates JH binding (Braun & Wyatt, 1996). Considering this potential JH‐binding function, a decrease in arylphorin subunit alpha‐like protein could be associated with a primer pheromone derepressed phenotype in fire ant virgin queens (Barker, 1978, 1979; Kearney, Toom, & Blomquist, 1977). Thus, fire ant queens may produce arylphorin subunit alpha‐like in the brain to prevent small levels of JH from triggering untimely reproductive development of alates. Taken together, a role of hexamerins as both storage and signaling molecules in fire ant queens is proposed, and as such was included under the conserved genetic toolkit (Table 4).

Whether expression changes of *hexamerin 1* occur in the brain or in surrounding fat cells, the changes in expression may have signaling significance in queens. Moreover, we showed that a JH mimic, S‐hydroprene, significantly decreased expression of *hexamerin 1* in whole bodies of virgin queens (Figure 6). This supports the idea that regulation of *hexamerin 1* is under control of JH, which titer increases with age in virgin queens (Brent & Vargo, 2003). This response to JH in virgins is inverse to what we previously showed for the vitellogenin receptor transcript level, which increases in the ovary of virgin queens upon application of a JH mimic (methoprene: Chen et al., 2004). Thus, JH increase in mature dealate virgins is associated with *hexamerin 1* downregulation, likely reflecting the use of hexamerin 1 protein stores for flight or egg production (vitellogenin synthesis) and with the increase in expression of the vitellogenin receptor transcript in the ovary (Chen et al., 2004).

### 4.3 Immunity

The mating process in itself can be damaging to the female insect, compromising a queen's cuticular line of defense (Kamimura, 2008); and founding of a colony exposes the queen to pathogens found in the nesting soil. Some ant species exhibit an increased immune response and become more pathogen‐tolerant after mating (Castella, Christe, & Chapuisat, 2009; Galvez & Chapuisat, 2014). In insect immune response, insect hemocytes and antimicrobial peptides (AMPs) play important roles in defense (Hillyer, 2016; Viljakainen & Pamilo, 2008; Vlisidou & Wood, 2015). We found upregulated genes in queen brains representative of these pathways; *astakine*, a neuropeptide, likely induces hematopoiesis (Lin, Kim, Lee, Soderhall, & Soderhall, 2009; Lin & Soderhall, 2011; Lin, Soderhall, & Soderhall, 2011) and *nc725*, which likely encodes an AMP (see below). This demonstrates multiple mechanisms of increased immune response in fire ant queens. This significant upregulation was present in the brain transcriptome of mated queens (Table 1, Figure 1). Astakines are related to vertebrate prokineticins and were described in a crustacean (crayfish) to induce hematopoiesis (Lin & Soderhall, 2011; Lin et al., 2009, 2011). Expression of *astakine* in a plant bug, *Lygus lineolaris*, increases after fungal infection (Shelby, Perera, & Snodgrass, 2015), supporting its immune role.

We found the gene *XR_850725* (*nc725*) currently annotated as a noncoding RNA, to be upregulated in mated queens (Table 1). Interestingly, *nc725* shares 100% identity with the transcript of the fire ant AMP gene *abaecin* (Casteels et al., 1990), which in the genome is located in the same region of the same contig as *nc725*. The *abaecin* gene is not currently annotated as such in the fire ant genome, and we propose that *nc725* encodes *abaecin*. Moreover, *abaecin* was previously identified as specifically present in mated polygyne queens in comparison with virgin alates in whole bodies utilizing a suppression subtractive hybridization approach and RT‐PCR and northern blot (Tian, Vinson, & Coates, 2004). The upregulation of the putative AMP *abaecin* is consistent with an increase in immune defense in mated queens that may be ecologically relevant under natural conditions as previously speculated (Tian et al., 2004). Our results with *abaecin* in the alate separation assay support that this gene is indeed upregulated by mating because there are no significant differences in its expression among virgins inhibited or disinhibited (Figure 4). However, we observed upregulation of *astakine* in virgin queens that dealated under queenless conditions (Figure 4). This suggests that at least *astakine* overexpression in dealate virgins may be mechanistically hardwired to changes in social context (dealation), older age (high nutritional status), or having achieved sexual maturation, but not strictly dependent of mating (Figure 4).

### 4.4 Signaling in queen maturation and postmating

In an effort to identify signaling molecules in queen brains, we performed transcriptomics and verified transcriptional expression by qRT‐PCR analyses of queens maintained under different conditions. Our research showed that the decrease in expression of hexamerins (*hexamerin 1* [Figures 1 and 2] and *arylphorin alpha*) is implicated in the transition from virgin alate to mated dealate but also, and for *hexamerin 1*, more significantly, in the transition of virgin alate to virgin dealate under queenless conditions (Figure 4), indicating that mating is associated with but not causative of these changes. This points to the role of hexamerins as nutritional signals or regulators of JH titer as discussed above. Brain dopamine levels are also associated with higher oogenesis and oviposition in virgin dealate queens (Boulay et al., 2001). Correspondingly, we found the *dopamine receptor 1* as the most abundant biogenic amine GPCR expressed in brain (Table 3), with *dopamine receptor 2* also highly expressed.

Mating affects female behavior and reproductive output, and numerous signals, endogenous or male‐transferred, are involved in these changes. In this context, we found the *tyramine receptor* highly expressed in queen brain, which is significant because of the discovery of male‐transferred tyramides during copulation (Chen & Grodowitz, 2017; Jones & Vander Meer, 2013). These tyramides may be processed to tyramine or other derivatives or may directly bind the tyramine receptor as shown in rats (Bunzow et al., 2001). Mating may also change tissue‐specific expression of GPCRs. We previously demonstrated protein expression of the sNPF receptor in brains of both virgin and mated queens but only in ovaries of mated queens, and in agreement, in this study, the transcripts for *sNPF receptor* and cognate peptide (*sNPY*) were also found highly expressed in brain (Table 3). It is accepted the sNPF system links nutrition and reproduction (Nagata, 2016), and therefore, we included it in the genetic conserved toolkit (Table 4).

## 5 CONCLUSIONS

Analyses of gene expression between dealate mated and alate virgin queen brain revealed changes in nutritional signaling/storage processes and upregulation of immune response; these changes may be key for successful reproduction. Interestingly, only 22 genes were differentially expressed between alate virgin and dealate mated queen brains. The low number of responsive genes raises the question; if major changes in gene expression are not occurring in the brain after mating, when do they occur? In which tissues are they expected to occur? Or, are only changes in protein expression significant? It is clear that virgin queen age and elevated JH titers are associated with ovarian readiness for vitellogenin uptake in the oocytes (Lu et al., 2009; Vargo, 1998).

One possibility is that changes in brain expression occur earlier in adult life (Nipitwattanaphon, Wang, Dijkstra, & Keller, 2013) and related to “maturation” and/or social context. In support of this, similar changes in expression were found for three GOIs in disinhibited virgin dealates similar to results of transcriptome comparisons of “mated” versus “virgin.” Therefore, it appears these changes in gene expression are driven, at least in part, by a switch in virgin queen physiological status. These changes were previously loosely referred to as either “maturation” (increase in weight and fat stores (Tschinkel, 1993) or “reproductive maturation” (changes occurring from adult eclosion to the age at which they engage in mating flights about 7–10 days old (Lofgren et al., 1975), and finally, “sexually immature virgin queens” were defined by light color of the cuticle and slender abdomen (Fletcher et al., 1983).

Broadly, our knowledge supports two inhibited virgin queen conditions with previtellogenic ovaries: “immature, inhibited queens” and “mature, inhibited queens.” Vitellogenin is present in the hemolymph of virgin queens; however, there is no significant uptake when they are inhibited by primer pheromone (Lewis et al., 2001). The “mature inhibited queens” may correspond to those that quickly dealated when separated from the influence of primer pheromone and developed their oocytes (Figure 5c) (Fletcher et al., 1983; Vargo & Laurel, 1994). These virgin queens could be considered reproductively mature (endocrinologically‐, age‐ and nutritionally competent) to engage in a mating flight. Lastly, a third category includes virgin queens actively engaging in a mating flight.

The graded nature of each queen maturation process may result in a continuum of gene expression changes that may be difficult to detect with those in mated queens when virgin queen physiological age is not taken into account, as gene expression in virgin queens changes from 1‐ to 11‐day posteclosion (Nipitwattanaphon et al., 2013). This likely contributed to the small number of DEGs herein identified. “Physiological age” then becomes a critical integrative factor to understand these transitions. Discovery of markers to unequivocally identify these physiological transitions will aid in dissecting the specific gene networks in signaling modules that respond to different endocrine, social, and environmental cues.

## CONFLICT OF INTEREST

None declared.

## AUTHOR CONTRIBUTIONS

TLC: designed and performed experiments, analyzed data, and wrote the paper. MEC: designed and performed experiments, analyzed data, and wrote methods. AKA: performed experiments, analyzed data, and wrote methods. CH: developed methodology, performed experiments, and wrote methods. CT: codirected research, designed experiments, analyzed data, and wrote the paper. PVP: directed research, designed experiments, analyzed data, and wrote the paper.

## DATA ACCESSIBILITY

Transcriptomic data (raw and processed data) were submitted to NCBI GEO (accession number GSE108063), under title “Fire ant alate virgin and dealate mated queen brain transcriptomes”). These data will be made public upon acceptance of the manuscript.
