# Supplementary material for: Brain gene expression analyses in virgin and mated queens of fire ants reveal mating‐independent and socially regulated changes
Source: Ecol Evol. 2018 Apr 2;8(8):4312–27. doi: 10.1002/ece3.3976 (PMC5916306; doi:10.1002/ece3.3976)
Supplement: Supplementary file 1 [file ECE3-8-4312-s001.pdf]

Supplementary Information
Supplementary Table 1. Primers utilized for qRT-PCR analyses

GOI	Accession number	Forward primer 5'-3'	Reverse Primer 5'-3'	Product (bp)
Hexamerin-like	XM_011157206	GGGTGTTACGGTCGAATCTG	TGAGCCTCCTTGTGACTGTG	108
Astakine-like	XM_011167741	ACGGATTATCCTGCGATTTC	CCGACTAGGTTCCACATAATCC	104
Noncoding RNA	XR_850725	CGATGATTTCTGCTCACGTC	ATCACCTTCGCCAAACATTC	103
Noncoding RNA	XR_850909	TGCAACCCAAACCACACTAA	TGATTGCCATGATTGTCAAGA	92
Elf1-Beta	XM_011169118	TCGCCAAGTCAAGTATCGTG	CCAAACAAGACCGTCCATTT	107
RPL18	XM_011165253	TACACCGACCACCGATTTCA	GATCACGGCGACGCAATT	80
Hexamerin 2	XM_011173560	CGTAGGATATGGACCGGTTG	GCCTTGATGTTGGATTTGCT	97
Arylphorin-Alpha	XM_011157183	GCCCTTTCCGTAGAGAGCTT	TGTTGACGTTGAAGCGGTAG	103
Arylphorin-Beta	XM_011157182	GCCGACTACAACACCATCAA	CGAAGGATTCACCAGAGAGG	108

Supplementary Table S2. Libraries statistics: number of reads mapping to the *Solenopsis invicta* genome for each replicate (Rep.).

	Alate queen brain		Mated queen brain	
	input	Mapped (% of input)	input	Mapped (% of input)
Rep1	13,665,083	12,464,405 (91.2)	17,305,195	16,128,864 (93.2)
Rep2	13,486,050	12,336,327 (91.5)	16,015,749	14,846,343 (92.7)
Rep3	14,717,332	13,368,742 (90.8)	18,335,231	17,143,926 (93.5)
Rep4	15,669,271	14,294,871 (91.2)	16,561,818	15,459,348 (93.3)

Supplementary Table 3: GPCRs expressed in virgin alate queen brain or mated dealate queen brain transcriptomes. GPCRs are annotated as currently identified in the *Solenopsis invicta* transcriptome and organized from highest to lowest average FPKM.

GeneInfo Identifier	Protein Accession	Transcript Accession	Locus	Current annotation	Alate FPKM	Mated FPKM
gi|751222462|	XP_011164467.1	XM_011166165	LOC105199190	rhodopsin	122.76	322.869
gi|751202946|	XP_011174320.1	XM_011176018	LOC105206526	dopamine receptor 1	77.9886	133.694
gi|751222464|	XP_011164468.1	XM_011166166	LOC105199191	rhodopsin-like	91.2739	77.2333
gi|751236330|	XP_011171996.1	XM_011173694	LOC105204568	PDF receptor	25.2926	51.0937
gi|751222675|	XP_011164585.1	XM_011166283	LOC105199276	probable G-protein coupled receptor AH9.1	28.8151	35.6691

gi|751209716|	XP_011157518.1	XM_011159216	LOC105194339	probable G-protein coupled receptor 52	25.6449	34.8058
gi|751223066|	XP_011164802.1	XM_011166501	LOC105199400	muscarinic acetylcholine receptor DM1 isoform X1	23.8323	28.1483
gi|751205599|	XP_011176389.1	XM_011178087	LOC105208274	tachykinin-like peptides receptor 99D, partial	17.3657	32.4827
gi|751223401|	XP_011164985.1	XM_011166683	LOC105199537	opsin, ultraviolet-sensitive-like	37.043	12.2558
gi|751238153|	XP_011173009.1	XM_011174707	LOC105205353	dopamine receptor 2-like	22.8932	21.386
gi|751212977|	XP_011159291.1	XM_011160989	LOC105195542	PREDICTED: G-protein coupled receptor moody isoform X2	17.3692	20.2122
gi|751212975|	XP_011159290.1	XM_011160988	LOC105195542	PREDICTED: G-protein coupled receptor moody isoform X1	20.1014	14.7491
gi|751232987|	XP_011170144.1	XM_011171842	LOC105203085	tachykinin-like peptides receptor 99D	12.7003	18.8504
gi|751223068|	XP_011164803.1	XM_011166500	LOC105199400	muscarinic acetylcholine receptor DM1 isoform X2	14.0267	11.4862
gi|751233286|	XP_011170315.1	XM_011172013	LOC105203239	allatostatin-A receptor-like isoform X1	13.6399	11.547
gi|751219449|	XP_011162809.1	XM_011164507	LOC105197902	calcitonin receptor-like	12.5495	12.6063
gi|751210001|	XP_011157676.1	XM_011159374	LOC105194460	diuretic hormone receptor-like	13.131	11.574
gi|751222422|	XP_011164446.1	XM_011166144	LOC105199174	5-hydroxytryptamine receptor 2A-like isoform X1	14.5434	10.0597
gi|751207421|	XP_011156273.1	XM_011157971	LOC105193512	tyramine receptor 1	9.84056	12.2824

gi|751223477|	XP_011165026.1	XM_011166724	LOC105199572	5-hydroxytryptamine receptor 2B-like	6.77122	14.5504
gi|751231536|	XP_011169333.1	XM_011171031	LOC105202476	octopamine receptor beta-1R-like	12.9282	6.29347
gi|751205401|	XP_011176282.1	XM_011177980	LOC105208194	uncharacterized protein LOC105208194	9.47505	9.49876
gi|751211218|	XP_011158340.1	XM_011160038	LOC105194900	5-hydroxytryptamine receptor 2A-like	7.82188	10.8045
gi|751206701|	XP_011155882.1	XM_011157580	LOC105193213	latrophilin Cirl-like	8.47064	8.25634
gi|751237973|	XP_011172906.1	XM_011174604	LOC105205262	probable muscarinic acetylcholine receptor gar-1	6.71836	9.72939
gi|751230013|	XP_011168496.1	XM_011170194	LOC105201914	alpha-1A adrenergic receptor-like	9.25873	6.82297
gi|751201048|	XP_011165260.1	XM_011166958	LOC105199739	probable G-protein coupled receptor No9	6.04333	8.33983
gi|751208599|	XP_011156916.1	XM_011158614	LOC105193922	tachykinin-like peptides receptor 86C, partial	6.81062	7.03414
gi|751218298|	XP_011162177.1	XM_011163875	LOC105197486	gastrin/cholecystokinin type B receptor-like isoform X2	6.27368	6.64
gi|751199635|	XP_011157701.1	XM_011159399	LOC105194469	tyramine receptor 1-like	6.55474	6.09929
gi|751212247|	XP_011158889.1	XM_011160587	LOC105195234	neuropeptides capa receptor-like	6.25855	6.23513
gi|751207849|	XP_011156508.1	XM_011158206	LOC105193663	histamine H2 receptor isoform X2	3.49217	8.65461

gi|751201367|	XP_011167017.1	XM_011168715	LOC105200910	opsin, blue-sensitive	5.64833	6.20141
gi|751211271|	XP_011158366.1	XM_011160064	LOC105194927	dopamine D2-like receptor	4.67986	6.46566
gi|751230111|	XP_011168550.1	XM_011170248	LOC105201947	adenosine receptor A2b-like isoform X1	5.87984	4.76843
gi|751204439|	XP_011175773.1	XM_011167349	LOC105200002	neuromedin-U receptor 2-like	5.06395	5.4427
gi|751224601|	XP_011165651.1	XM_011167349	LOC105200002	neuromedin-U receptor 2-like	5.06395	5.4427
gi|751214247|	XP_011159970.1	XM_011161668	LOC105195979	FMRFamide receptor isoform X2	3.61381	6.12039
gi|751236000|	XP_011171810.1	XM_011173508	LOC105204438	uncharacterized protein LOC105204438	3.49725	6.16527
gi|751233288|	XP_011170316.1	XM_011172014	LOC105203239	allatostatin-A receptor-like isoform X1	5.06126	4.10991
gi|751218178|	XP_011162112.1	XM_011163810	LOC105197452	orexin receptor type 1-like	3.81174	5.29119
gi|751238033|	XP_011172940.1	XM_011174638	LOC105205294	tachykinin-like peptides receptor 86C, partial	4.35839	3.8324
gi|751206733|	XP_011155898.1	XM_011157596	LOC105193226	trace amine-associated receptor 9	4.70743	3.447
gi|751224717|	XP_011165717.1	XM_011167415	LOC105200046	gastrin-releasing peptide receptor-like	3.32243	4.03507
gi|751205799|	XP_011155394.1	XM_011157092	LOC105192838	cholecystokinin receptor-like	3.71336	3.47172
gi|751225754|	XP_011166274.1	XM_011167972	LOC105200419	octopamine receptor beta-2R isoform X1	2.99628	3.91515
gi|751217754|	XP_011161886.1	XM_011163584	LOC105197287	octopamine receptor beta-3R-like	2.60488	3.37885

gi|751202675|	XP_011173067.1	XM_011174765	LOC105205389	gonadotropin-releasing hormone II receptor isoform X2	1.65061	4.11409
gi|751207336|	XP_011156229.1	XM_011157927	LOC105193474	octopamine receptor beta-3R-like	2.15744	3.39361
gi|751220611|	XP_011163444.1	XM_011165142	LOC105198435	FMRFamide receptor-like	3.1209	2.29737
gi|751232028|	XP_011169609.1	XM_011171307	LOC105202687	neuropeptide FF receptor 2	1.93127	2.68557
gi|751206248|	XP_011155636.1	XM_011157334	LOC105193015	protocadherin-like wing polarity protein stan	1.99164	2.43573
gi|751224894|	XP_011165811.1	XM_011167509	LOC105200112	growth hormone secretagogue receptor type 1	2.2874	1.8629
gi|751208123|	XP_011156656.1	XM_011158354	LOC105193776	lutropin-choriogonadotropic hormone receptor	2.1355	1.84212
gi|751212866|	XP_011159228.1	XM_011160926	LOC105195500	prolactin-releasing peptide receptor-like	1.74498	2.1542
gi|751214243|	XP_011159967.1	XM_011161665	LOC105195979	FMRFamide receptor isoform X1	2.22963	1.61088
gi|751225756|	XP_011166275.1	XM_011167973	LOC105200419	octopamine receptor beta-2R isoform X2	2.15962	1.58662
gi|751224703|	XP_011165709.1	XM_011167407	LOC105200038	LOW QUALITY PROTEIN: neuromedin-B receptor-like	2.19548	1.50736
gi|751236288|	XP_011171973.1	XM_011173671	LOC105204554	secretin receptor-like isoform X2	1.80764	1.47041
gi|751205399|	XP_011176281.1	XM_011177978	LOC105208194	uncharacterized protein LOC105208194	1.74897	1.40877

gi|751204377|	XP_011175739.1	XM_011177437	LOC105207791	neuropeptides capa receptor-like	1.38895	1.70664
gi|751199398|	XP_011156359.1	XM_011158057	LOC105193552	prostaglandin E2 receptor EP3 subtype	1.73066	1.25177
gi|751202673|	XP_011173059.1	XM_011174757	LOC105205389	gonadotropin-releasing hormone II receptor isoform X1	1.0036	1.87529
gi|751210368|	XP_011157876.1	XM_011159574	LOC105194591	allatostatin-A receptor-like	1.52128	1.32521
gi|751217576|	XP_011161788.1	XM_011163486	LOC105197213	neuropeptides capa receptor-like isoform X2	1.63435	1.16905
gi|751202512|	XP_011172409.1	XM_011174107	LOC105204861	allatostatin-A receptor	1.59364	1.14899
gi|751205345|	XP_011176251.1	XM_011177949	LOC105208166	protein trapped in endoderm-1	1.51694	1.10737
gi|751222424|	XP_011164447.1	XM_011166145	LOC105199174	5-hydroxytryptamine receptor 2A-like isoform X2	0.000951	2.46655
gi|751234791|	XP_011171145.1	XM_011172843	LOC105203919	uncharacterized protein LOC105203919	1.27474	1.18695
gi|751212979|	XP_011159292.1	XM_011160990	LOC105195543	G-protein coupled receptor moody	1.24731	1.17934
gi|751212250|	XP_011158890.1	XM_011160588	LOC105195236	neuropeptides capa receptor-like	1.88112	0.468675
gi|751212870|	XP_011159230.1	XM_011160929	LOC105195500	prolactin-releasing peptide receptor-like	0.676592	1.60495
gi|751236286|	XP_011171972.1	XM_011173670	LOC105204554	secretin receptor-like isoform X1	0.805082	1.36281
gi|751199777|	XP_011158500.1	XM_011160198	LOC105195005	relaxin receptor 1	1.34767	0.788181

gi|751221554|	XP_011163962.1	XM_011165660	LOC105198818	alpha-2A adrenergic receptor	0.988972	1.14645
		XR_850821	LOC105197486	gastrin/cholecystokinin type B receptor-like (LOC105197486), transcript variant X2, misc_RNA	0.130718	1.98553
gi|751236292|	XP_011171975.1	XM_011173673	LOC105204554	secretin receptor-like isoform X4	0.991241	1.06065
gi|751236290|	XP_011171974.1	XM_011173672	LOC105204554	secretin receptor-like isoform X3	1.58782	0.348021
gi|751204965|	XP_011176051.1	XM_011177749	LOC105208023	green-sensitive opsin-like	0.330279	1.35461
gi|751212868|	XP_011159229.1	XM_011160928	LOC105195500	prolactin-releasing peptide receptor-like	1.67119	0.00067
gi|751203152|	XP_011175066.1	XM_011176764	LOC105207336	cardioacceleratory peptide receptor-like	0.592684	0.971376
gi|751236282|	XP_011171969.1	XM_011173667	LOC105204554	secretin receptor-like isoform X1	0.597832	0.886539
gi|751212872|	XP_011159231.1	XM_011160927	LOC105195500	prolactin-releasing peptide receptor-like	0.000375	1.4159
gi|751214494|	XP_011160104.1	XM_011161802	LOC105196073	LOW QUALITY PROTEIN: probable G-protein coupled receptor B0563.6	0.529527	0.875558
gi|751209558|	XP_011157431.1	XM_011159129	LOC105194275	gonadotropin-releasing hormone receptor-like	0.330771	0.972118
gi|751224902|	XP_011165816.1	XM_011167514	LOC105200117	thyrotropin-releasing hormone receptor-like	0.349814	0.849813

gi|751236194|	XP_011171921.1	XM_011173619	LOC105204531	G-protein coupled receptor Mth2-like	0.508774	0.580146
gi|751228090|	XP_011167439.1	XM_011169137	LOC105201199	adenosine receptor A2b-like	0.391874	0.666697
gi|751236294|	XP_011171976.1	XM_011173674	LOC105204554	secretin receptor-like isoform X5	0.545303	0.399329
gi|751228088|	XP_011167438.1	XM_011169136	LOC105201199	adenosine receptor A2b-like	0.381256	0.409619
gi|751232585|	XP_011169919.1	XM_011171617	LOC105202925	5-hydroxytryptamine receptor 1-like	0.222941	0.555203
gi|751218295|	XP_011162176.1	XM_011163874	LOC105197486	gastrin/cholecystokinin type B receptor-like isoform X1	0.34228	0.164363
gi|751236280|	XP_011171968.1	XM_011173666	LOC105204554	secretin receptor-like isoform X1	0	0.484745
gi|751234850|	XP_011171180.1	XM_011172878	LOC105203942	gonadotropin-releasing hormone receptor-like, partial	0.456537	0
gi|751225429|	XP_011166100.1	XM_011167798	LOC105200319	probable G-protein coupled receptor Mth-like 2	0.21404	0.034813
gi|751205397|	XP_011176280.1	XM_011177979	LOC105208194	uncharacterized protein LOC105208194	0.171619	0
gi|751225427|	XP_011166099.1	XM_011167797	LOC105200318	uncharacterized protein LOC105200318	0.036088	0.068215
gi|751225752|	XP_011166273.1	XM_011167971	LOC105200419	octopamine receptor beta-2R isoform X1	0.002057	0.098628
gi|751214816|	XP_011160284.1	XM_011161982	LOC105196189	uncharacterized protein LOC105196189	0	0.098093

gi|751213593|	XP_011159613.1	XM_011161311	LOC105195745	vasopressin V1a receptor-like isoform X2	0.075512	0
gi|751213591|	XP_011159612.1	XM_011161310	LOC105195745	vasopressin V1a receptor-like isoform X1	0	0.033531
gi|751203577|	XP_011175311.1	XM_011177009	LOC105207504	uncharacterized protein LOC105207504 isoform X4	0	0.027589
gi|751205347|	XP_011176253.1	XM_011177951	LOC105208166	protein trapped in endoderm-1	0.002619	0.000177
gi|751207847|	XP_011156507.1	XM_011158205	LOC105193663	histamine H2 receptor isoform X1	0	0.002322
gi|751199633|	XP_011157692.1	XM_011159390	LOC105194469	tyramine receptor 1-like	0.0005	0.001214
gi|75123011 3	XP_011168551.1	XM_011170249	LOC105201947	adenosine receptor A2b-like isoform X2	0.000606	0.000314
gi|751214241|	XP_011159966.1	XM_011161664	LOC105195979	FMRFamide receptor isoform X1	0.000179	0
gi|751217574|	XP_011161787.1	XM_011163485	LOC105197213	neuropeptides capa receptor-like isoform X1	3.14E-05	8.00E-05
gi|751236196|	XP_011171922.1	XM_011173620	LOC105204531	G-protein coupled receptor Mth2-like	7.72E-05	2.01E-05
gi|751209556|	XP_011157430.1	XM_011159128	LOC105194275	gonadotropin-releasing hormone receptor-like	6.94E-06	0
gi|75120357 5	XP_011175310.1	XM_011177008	LOC105207504	G-protein coupled receptor Mth2-like isoform X3	0	4.18E-06
gi|75120357 3	XP_011175309.1	XM_011177007	LOC105207504	uncharacterized protein LOC105207504 isoform X2	0	2.33E-06

gi|751203571	XP_011175308.1	XM_011177006	LOC105207504	G-protein coupled receptor Mth2-like isoform X1	0	5.29E-07
gi|751203569	XP_011175307.1	XM_011177005	LOC105207504	G-protein coupled receptor Mth2-like isoform X1	0	4.22E-07
gi|751209550|	XP_011157427.1	XM_011159125	LOC105194275	gonadotropin-releasing hormone receptor-like	0	0
gi|751209552|	XP_011157428.1	XM_011159126	LOC105194275	gonadotropin-releasing hormone receptor-like	0	0
gi|751209554|	XP_011157429.1	XM_011159127	LOC105194275	gonadotropin-releasing hormone receptor-like	0	0
gi|751217578|	XP_011161789.1	XM_011163487	LOC105197213	neuropeptides capa receptor-like isoform X3	0	0
gi|751202671|	XP_011173053.1	XM_011174751	LOC105205389	gonadotropin-releasing hormone II receptor isoform X1	0	0
gi|751214245|	XP_011159968.1	XM_011161666	LOC105195979	FMRFamide receptor isoform X1	0	0
gi|751236274|	XP_011171965.1	XM_011173663	LOC105204554	secretin receptor-like isoform X1	0	0
gi|751236276|	XP_011171966.1	XM_011173664	LOC105204554	secretin receptor-like isoform X1	0	0
gi|751236278|	XP_011171967.1	XM_011173665	LOC105204554	secretin receptor-like isoform X1	0	0

gi|751236284|	XP_011171971.1	XM_011173669	LOC105204554	secretin receptor-like isoform X1	0	0
gi|751206087|	XP_011155552.1	XM_011157250	LOC105192960	gonadotropin-releasing hormone receptor-like	0	0
gi|751239722|	XP_011173862.1	XM_011175560	LOC105206029	glucagon receptor-like, partial	0	0
gi|751227496|	XP_011167228.1	XM_011168926	LOC105201068	uncharacterized protein LOC105201068, partial	0	0
gi|751219652|	XP_011162921.1	XM_011164619	LOC105197996	uncharacterized protein LOC105197996	0	0
gi|751234416|	XP_011170936.1	XM_011172634	LOC105203747	somatostatin receptor type 2-like		

Supplementary methods. Analyses of hexamerins expression in the head and thorax/abdomen of queens

For comparisons of gene expression of four different hexamerins between head and thorax plus abdomen (Fig. S2), alate virgin queens were decapitated with fine forceps. Ten queens were used per independent replicate immediately placing either heads or thorax/abdomens into 100 µL Trizol on ice. Samples were stored at -80˚C overnight and thawed on ice. Tissues were homogenized in microcentrifuge tubes using sterile plastic pestles (Thermo Fisher Scientific), after which RNA was extracted and cDNA was synthesized as described.

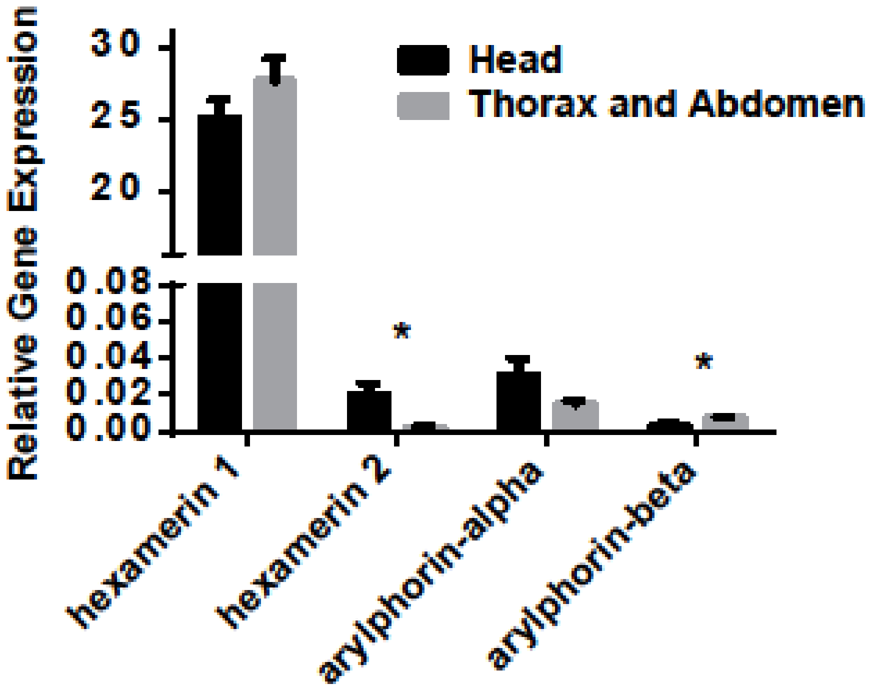

Supplemental Figure 1: Expression of hexamerins in virgin alate queen heads and thorax/ abdomens. Gene expression determined by qPCR was normalized to both Elf1-Beta and RPL18. Bars indicate mean relative gene expression (ΔCt) +/- SEM, n = 4. "*" indicate a significant difference ($p \leq 0.05$) for the examined corresponding hexamerins between heads and thorax/abdomen, as determined by *t*-tests. Hexamerin 1, $p = 0.2590$; Hexamerin 2, $p = 0.0226$; Arylphorin-alpha, $p = 0.1707$; Arylphorin-beta, $p = 0.0254$.
